# Hyponatremia in Severe Malaria: Evidence for an Appropriate Anti-diuretic Hormone Response to Hypovolemia

**DOI:** 10.4269/ajtmh.2009.08-0393

**Published:** 2009-01

**Authors:** Josh Hanson, Amir Hossain, Prakaykaew Charunwatthana, Mahtab Uddin Hassan, Timothy M. E. Davis, Sophia W. K. Lam, S. A. Paul Chubb, Richard J. Maude, Emran Bin Yunus, Gofranul Haque, Nicholas J. White, Nicholas P. J. Day, Arjen M. Dondorp

**Affiliations:** Mahidol-Oxford Tropical Medicine Research Unit, Faculty of Tropical Medicine, Mahidol University, Bangkok, Thailand; Chittagong Medical College Hospital, Chittagong, Bangladesh; School of Medicine and Pharmacology, Fremantle Hospital, Fremantle, Western Australia, Australia; Department of Clinical Biochemistry and School of Medicine and Pharmacology, Fremantle Hospital, Fremantle, Western Australia, Australia; Centre for Tropical Medicine, Nuffield Department of Clinical Medicine, John Radcliffe Hospital, University of Oxford, Oxford, United Kingdom

## Abstract

Although hyponatremia occurs in most patients with severe malaria, its pathogenesis, prognostic significance, and optimal management have not been established. Clinical and biochemical data were prospectively collected from 171 consecutive Bangladeshi adults with severe malaria. On admission, 57% of patients were hyponatremic. Plasma sodium and Glasgow Coma Score were inversely related (*r_s_* = −0.36, *P* < 0.0001). Plasma antidiuretic hormone concentrations were similar in hyponatremic and normonatremic patients (median, range: 6.1, 2.3–85.3 versus 32.7, 3.0–56.4 pmol/L; *P* = 0.19). Mortality was lower in hyponatremic than normonatremic patients (31.6% versus 51.4%; odds ratio [95% confidence interval]: 0.44 [0.23–0.82]; *P* = 0.01 by univariate analysis). Plasma sodium normalized with crystalloid rehydration from (median, range) 127 (123–140) mmol/L on admission to 136 (128–149) mmol/L at 24 hours (*P* = 0.01). Hyponatremia in adults with severe malaria is common and associated with preserved consciousness and decreased mortality. It likely reflects continued oral hypotonic fluid intake in the setting of hypovolemia and requires no therapy beyond rehydration.

## Introduction

Hyponatremia is observed in the majority of adult and pediatric patients with severe malaria.^1^,^2^ A number of underlying mechanisms have been proposed, including the administration of hypotonic fluids, the syndrome of inappropriate anti-diuretic hormone secretion (SIADH),^3^–^5^ cerebral salt wasting (CSW),^6^ losses through sweat and the gastrointestinal tract,^7^ renal losses,^8^ and the “sick cell syndrome.”^9^ There is, however, no consensus as to their relative contributions.^6^,^10^ An understanding of the pathophysiology is of major clinical importance because it might direct management strategies. In a patient with SIADH, for example, fluid restriction is the cornerstone of treatment.^11^ By contrast, treatment of CSW requires large volumes of replacement fluid.^12^

The importance of correcting the hyponatremia that complicates severe malaria is debated. Some authors, noting that hyponatremia is associated with adverse outcomes in other clinical situations,^13^ have suggested that it should be specifically and aggressively treated.^6^ Others have argued that it is a physiological response to hypovolemia requiring no specific therapy beyond rehydration,^2^ although the extent of hypovolemia in patients with severe malaria remains controversial.^9^,^14^

In this study, we assessed the pathogenesis and prognosis of malaria-associated hyponatremia, using prospectively collected data and plasma samples from two intervention studies in adult patients with severe falciparum malaria.

## Materials and Methods

### Patients.

Patients enrolled in clinical trials conducted at Chittagong Medical College Hospital (CMCH), Bangladesh, between 2003 and 2007, were included in this study. CMCH is a 1,000-bed tertiary referral hospital in the country's second largest city. There is no malaria transmission in the city of Chittagong, but CMCH serves a wide area including the Chittagong Hill Tracks, where malaria is unstable with seasonal transmission. Informed consent was obtained from the families of all patients enrolled in the trials, which had been approved by the Ethics Committee of the Ministry of Health, Bangladesh, and OXTREC, the Ethical Committee for studies in tropical countries of Oxford University, UK. Patients from 2003 to 2005 were enrolled in a trial examining the utility of *N*-acetylcysteine as adjuvant therapy in severe malaria (unpublished data). In 2006 and 2007, the patients were from a trial examining the efficacy of levamisole as adjuvant therapy in severe malaria (study ongoing). All patients had to satisfy pre-specified criteria for severe malaria^15^ and have asexual forms of *Plasmodium falciparum* on a peripheral blood film.

### Clinical procedures.

Baseline venous blood was collected at study entry. Urine was collected, where possible, by indwelling catheter. Thick and thin film microscopy was performed immediately to confirm eligibility, and basic laboratory results (plasma sodium, plasma potassium, blood urea nitrogen, blood glucose, plasma bicarbonate, plasma pH, hemoglobin, and hematocrit) were available within minutes through the use of a portable hand-held point-of-care analyzer (iStat with EC8 + cartridges; Abbott Laboratories, Abbott Park, IL). Additional plasma was stored in liquid nitrogen until assayed for anti-diuretic hormone (ADH) and brain natriuretic peptide (BNP). Urine specimens were frozen until subsequent analysis of electrolyte concentrations. Patients were managed according to published WHO guidelines,^7^,^16^ including resuscitation with normal saline titrated against the clinical assessment of dehydration.

ADH concentrations were estimated using a radioimmunoassay of extracted plasma (Buhlmann Laboratories, Basel, Switzerland). N-terminal fragment BNP (NT-pro BNP) was measured using reagents supplied by Roche Diagnostics on an Elecsys 2010 analyzer (Roche Diagnostics, Castle Hill, Australia). The normal ranges were 0.1–7.0 pmol/L for ADH and < 97 ng/L for NT-pro BNP.

### Data analysis.

The Cockcroft and Gault formula^17^ was used to estimate creatinine clearance as a measure of glomerular filtration.^18^ Plasma osmolarity was calculated using the formula 2 × (plasma Na) + (plasma blood urea nitrogen) + plasma glucose (all in SI units; reference range, 275–295 mosm/kg). Fractional excretion of sodium (FENa) was calculated as (urinary sodium × plasma creatinine)/(plasma sodium × urinary creatinine) × 100.

Patient data were de-identified, entered into a computerized database (Microsoft Access; Microsoft Corp., Redmond, WA), and analyzed using a statistical software package (Stata 9.0; StataCorp, College Station, TX). Unless otherwise indicated, data are presented as mean and 95% confidence intervals (CIs). Variables that were not normally distributed were analyzed using Spearman rho and Wilcoxon signed rank test. Fisher exact and χ^2^ tests were used to assess dichotomous variables. Logistic regression was used to identify independent predictors of outcome.

## Results

### Baseline characteristics.

One hundred eighty-one patients (median age, 35 years; range, 13–70 years; 79.5% male) were recruited to studies at CMCH over 5 years. Of the 181 patients, 171 (94.5%) had sufficient data for these analyses. Of the 10 patients not included, 5 did not have an iStat specimen collected, 4 had insufficient admission clinical data, and 1 had an invalid iStat result. The characteristics of the included patients are recorded in Table 1.

### Prevalence and correlates of hyponatremia.

The mean admission plasma sodium was 133 mmol/L (range, 117–155 mmol/L); 57% were hyponatremic (plasma sodium < 135 mmol/L) and 30% had a plasma sodium < 130 mmol/L. Fifty-four (35%) patients received intravenous fluid (usually < 1 L) before enrolment, and in 17 cases, it was unclear whether fluid had been administered. If these 71 patients are excluded, the mean (95% CI) plasma sodium [133 mmol/L (131–134 mmol/L)] and the proportion of patients with a plasma sodium < 135 and 130 mmol/L (61% and 31%, respectively) remained similar. For the purposes of this study, data from these patients were not analyzed separately.

**Figure 1.**
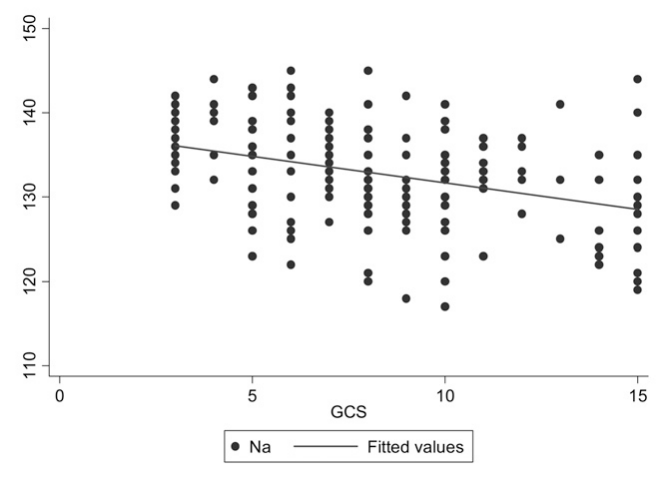
Plasma sodium versus Glasgow Coma Score on admission.

Compared with patients with a normal plasma sodium (135–145 mmol/L), those with hyponatremia had a higher Glasgow Coma Scores (GCSs), had greater parasite densities, and were less acidotic (Table 2). There was no significant difference in the admission creatinine clearance between the two groups. When the data were pooled, there was an inverse relationship between GCS and the admission plasma sodium (*N* = 171, *r*_s_ = −0.36, *P* < 0.0001; Figure 1).

There was no relationship between the plasma sodium and the urinary sodium measured either as the urinary sodium concentration (*r*_s_ = 0.1, *P* = 0.17) or FENa (*r*_s_ = 0.09, *P* = 0.26). Sixty-seven patients had their plasma osmolarity measured and thus could have an osmolar gap calculated. In these patients, there was no relationship between the plasma sodium and the measured osmolar gap (*r*_s_ = −0.13, *P* = 0.29).

### Plasma ADH, BNP, and hyponatremia.

Plasma ADH was measured using the admission blood sample from 30 patients (all those from 2006). Patients who were hyponatremic (*N* = 24) had a median (range) ADH of 6.1 (2.3–85.3) pmol/L compared with 32.7 (3.0–56.4) pmol/L in those with a normal plasma sodium level (*N* = 6; *P* = 0.19). There was a significant relationship between plasma ADH levels and the measured plasma osmolarity (*r*_s_ = 0.39, *N* = 30, *P* = 0.03). Plasma ADH was detected in all eight patients with a calculated osmolarity of < 280 mosm/kg (range, 2.3–40.1 pmol/L).

Twenty patients from the 2006 cohort had plasma BNP measured. There was no relationship between BNP and plasma sodium (*r*_s_ = 0.13, *P* = 0.58). Hyponatremic patients (*N* = 17) had a median (range) BNP of 520 (19–8,899) pg/mL compared with 610 (374–869) pg/mL in patients with a normal plasma sodium (*N* = 3). There was no relationship between BNP and urinary sodium (*r*_s_ = 0.21, *P* = 0.38) or FENa (*r*_s_ = 0.31, *P* = 0.19).

### Plasma sodium and outcome.

The mortality rate in the 171 patients was 40.4%. The 102 patients who survived had a lower admission plasma sodium [132 (130–133) mmol/L] than the 69 fatal cases [134 (133–136) mmol/L; *P* = 0.01]. Significantly fewer patients who were hyponatremic on admission died than those with a normal plasma sodium (odds ratio [95% CI]; 0.44 [0.23–0.82], *P* = 0.01). In addition, fewer patients with hyponatremia developed pulmonary edema, although the requirement for dialysis was similar (Table 2). In a logistic regression model with death as the dependent variable and hyponatremia and coma (GCS < 11) as independent variables, hyponatremia was not independently associated with outcome (OR, 0.54; 95% CI, 0.28–1.06; *P* = 0.07), but coma remained a strong associate (OR, 5.8; 95% CI, 2.1–15.9; *P* = 0.001).

Twelve patients had sequential specimens available for analysis (all those patients who survived to discharge and had complete specimen collection in 2006; Table 4) Two patients received dialysis on the first day of admission, affecting the interpretation of the serial measurements, and so their data were removed from the analysis. Hyponatremia improved rapidly in the remaining 10 patients with rehydration with normal saline. The median plasma sodium in these patients was 127 mmol/L (range, 123–140 mmol/L) on admission and 136 mmol/L (range, 128–149 mmol/L) at 24 hours (*P* = 0.01). Creatinine clearance and plasma osmolarity also normalized during treatment, whereas the urine sodium concentrations and FENa rose (data not shown).

## Discussion

This study confirmed that hyponatremia is common in adults with severe malaria. Although there are a number of potential causes,^7^ our data suggest that the major causative factor was ADH released appropriately in response to the hypovolemia evident clinically in the majority of patients at presentation. A decreased effective circulating blood volume will overcome the inhibition of ADH secretion that results from decreased plasma osmolarity.^19^ Indeed, plasma ADH was easily measurable in patients with a plasma osmolarity < 280 mosm/kg when there should no longer be a stimulus for ADH release.^20^

Under these circumstances, a hypovolemic but conscious patient who continues to drink water (or another hypotonic fluid) will develop a dilutional hyponatremia. Our data showed an inverse correlation between level of consciousness and plasma sodium. It is therefore unsurprising that hyponatremic patients as a group had a better outcome than those with a normal plasma sodium, because impaired consciousness has repeatedly been shown to be a strong predictor of mortality in severe malaria (see Table 3).^21^,^22^ The fact that the association between hyponatremia and survival was no longer significant when controlled for impaired consciousness supports our hypothesis. Our data do not suggest that there is an important role for the SIADH in the development of malaria-associated hyponatremia. After admission, patients were rehydrated with intravenous normal saline—rather than being fluid restricted—and hyponatremia resolved with other markers of hypovolemia including creatinine clearance, the urea:creatinine ratio, and the FENa. Although the exact amounts of administered fluid were not recorded, patients without pulmonary edema usually received between 2 and 5 L of normal saline in the first 24 hours.

Other proposed explanations for the hyponatremia associated with severe malaria also seem inconsistent with our data. The patients were hyponatremic on admission, mostly before any active rehydration, thus excluding an important contribution from hypotonic intravenous fluid administration. The absence of an association with the urinary sodium concentration and FENa argues against renal salt handling being the primary explanation for hyponatremia. Indeed, there was no association between hyponatremia and renal impairment. The inverse association with the GCS suggests that CSW, an entity that is itself controversial,^23^ is not responsible for hyponatremia. Sweat and gastrointestinal secretions are hypotonic,^24^,^25^ and thus if losses from these sources were significant, patients would be hypernatraemic and hyperosmolar. The “sick cell” syndrome proposes that, in severe illness, patients develop increased cell membrane permeability with leakage of intracellular solutes and an osmotic gradient that leads to the passage of water from the cell and a resultant dilutional hyponatremia. Some authors have proposed this as a mechanism for hyponatremia in malaria,^9^ but our data did not show a relationship between plasma sodium and osmolar gap.

Data supporting our conclusions come from studies in adults and children. Sitprija and others^26^ examined the volume status and hemodynamic responses of a series of Thai adults with moderately severe malaria. These authors identified a group of patients with reduced mean arterial pressure and creatinine clearance who also had activation of the renin–angiotensin system and sympathetic nervous system and release of ADH. These patients were relatively hyponatremic. The authors hypothesized that these findings could be explained by a reduced effective circulating volume secondary to systemic vasodilatation, which led, in turn, to ADH release and a dilutional hyponatremia. English and others,^2^ in a study of African children with severe malaria, found a statistically significant association between weight gain and plasma sodium. Hyponatremic patients gained a mean of 2.4% body weight compared with a mean of 4.3% in the normonatremic group, suggesting that the hyponatremic patients were less dehydrated as a result of water retained by appropriate rather than inappropriate ADH secretion.

A small study of imported malaria in the United Kingdom showed no relationship between plasma ADH concentrations and plasma sodium, but there were no data presented regarding disease severity.^27^ A small study performed on a German intensive care unit concluded that SIADH contributes to the pathogenesis of hyponatremia in severe malaria, because plasma ADH concentrations were higher than expected for the plasma sodium concentration.^3^ However, despite the fact that invasive monitoring was available, data were not corrected for hypovolemia, a factor that needs to be excluded before SIADH can be diagnosed.^20^ A study in Kenyan children with severe malaria^28^ suggested that 67% of hyponatremic cases had evidence of SIADH, based on the presence of detectable ADH levels despite low plasma sodium concentrations. However, because the patients were dehydrated and gained weight with intravenous rehydration, hypovolemia as a drive for ADH secretion cannot be excluded. Serial plasma electrolyte and ADH concentration data were collected but not reported. It would have been interesting to observe changes in plasma sodium and ADH with rehydration. Cases of true SIADH in patients with severe falciparum malaria have been reported^29^ but are likely to be uncommon.

Our study had limitations. Simultaneous longitudinal measurements of plasma and urine biochemistry, ADH concentrations, and more invasive measurements of volume status, showing a simultaneous fall in ADH and rise in plasma sodium with rehydration, would have strengthened our observations. Our study was performed in a resource-poor, tropical setting where patients tend to present late, having had very little health care before admission. It examined only adult patients. The generalizability of our findings to other populations, especially those in non-tropical settings and children, may be limited.

In conclusion, this study showed that hyponatremia is a common feature in adults presenting with severe malaria but is not associated with increased mortality. Hypovolemia, and not SIADH, is the likely major pathophysiological mechanism, and simple rehydration with normal saline restores the plasma sodium to the normal range in the majority of cases.

## Figures and Tables

**Table 1:** Patient characteristics

Median age (years)	35 (30–35)
Male sex (%)	136/171 (79.5%)
Mean mean arterial pressure (mmHg)	81 (79–84)
Mean pulse rate (beats/min)	115 (109–121)
Mean respiratory rate (breaths/min)	32 (31–33)
Mean oxygen saturation (%)	95 (94–95)
Mean plasma glucose (mmol/L)	8.6 (7.7–9.5)
Median Glasgow Coma Score	8 (7–9)
Mean sodium (mmol/L)	133 (132–134)
Mean potassium (mmol/L)	4.38 (4.23–4.52)
Mean plasma urea (mmol/L)	19.0 (16.9–21.1)
Mean plasma creatinine (µmol/L)	167 (146–189)
Mean plasma bicarbonate (mmol/L)	16 (15.7–17.2)
Mean base excess	−8.3 (−9.3 to −7.3)
Mean venous lactate (mmol/L)	5.8 (5.3–6.4)
Mean total bilirubin (µmol/L)	134 (110–158)
Mean direct bilirubin (µmol/L)	68 (52–84)
Median peripheral parasite count (%)	2.55 (1.9–3.4)
Mean hematocrit	29.7 (28.4–31.1)
Median plasma Anti-diuretic hormone (pmol/L)	7.45 (5–22.6)
Median plasma brain natriuretic peptide (pg/mL)	554 (391–1,678)
Mean fractional excretion of sodium	1.16 (0.93–1.4)
Mean calculated osmolality (mosm/L)	293 (290–297)
Mean urinary osmolality (mosm/L)	528 (500–557)
Coma on admission*	130/171 (76%)
Acute renal failure†	56/171 (32.7%)
Developed Pulmonary edema during admission	21/169 (12%)
Severe anemia‡	20/171 (11.7%)
Spontaneous bleeding	7/171 (4%)
Convulsions	26/161 (16%)
Hypoglycemia§	5/171 (3%)
Shock¶	9/170 (5.3%)
Died	69/171 (40.4%)

All values are means (95% CI) and are on admission unless otherwise stated.

* Glasgow Coma Score < 11.

† BUN on admission > 21.4 mmol/L (60 mg/dL).

‡ Hematocrit < 20 on admission.

§ Blood glucose level < 2 mmol/L.

¶ Systolic blood pressure < 80 mm of Hg and cool peripheries.

**Table 2:** Comparison of hyponatremic patients and patients with a normal plasma sodium*

	Plasma Na < 135 (*N* = 98)	Na normal* (*N* = 70)	*P* value
GCS	9 (8–10)	6 (6–7)	0.0002
Peripheral parasite count (%)	4 (2.7–5.6)	1.5 (1−2.1)	0.0008
Base excess	−7 (−8 to −5)	−10 (−11.1 to −5.9)	0.18
Plasma bicarbonate (mmol/L)	18.2 (16.7–19.3)	14.9 (13.9–17)	0.01
Creatinine clearance (mL/min/1.73 m^2^)	62.1 (50.3–73.1)	50 (39–71)	0.18
Required dialysis	19/98 19.4%	15/70 21.4%	0.85
Pulmonary edema developed	6/97† 6.2%	14/69† 20.3%	0.008
Died	31/98 (31.6%)	36/70 (51.4%)	0.01

All values are medians (range).

* Normal plasma sodium 135–145 mmol/L.

† In one patient in each group, it was not possible to determine whether pulmonary edema developed.

**Table 3:** Comparison of patients with normal and impaired GCS

	GCS ≥3; 15 (*N* = 18)	GCS < 15 (*N* = 153)	*P* value
Na (mmol/L)	127 (124–132)	134 (132–135)	0.006
Creatinine clearance (mL/min)	77.2 (47–101.6)	54 (45.3–64.6)*	0.06
Required dialysis	2/18 (11.1%)	32/153 (20.9%)	0.53
HCO_3_ (mmol/L)	18.8 (17.1–20.1)	16 (14.5–17)	0.048
Base excess	−5 (−7 to −3)	−8 (−10 to −6)	0.054
Death	2/18 (11.1%)	67/153 (44.2%)	0.009
Osmolarity (mosm/kg)	275 (264–290)	293 (288–397)	0.002
Lactate (mmol/L)	3.5 (3–4.6)	4.9 (4.4–5.5)	0.01
Parasite count (%)	11.3 (5–14.6)	2.1 (1.6–3)	0.001
Clinical hydration†	Mild	Moderate	0.08
ADH†	3.85 (2.32–6.34)	11.1 (4.9–34.5)	0.02

All values are medians (range).

* Six did not have a plasma creatinine on admission.

† Data only available for 30 patients in 2006.

**Table 4:** Plasma and urine biochemistry in patients with sequentially collected specimens

	Admission	24 hours	Discharge
Plasma sodium (mmol/L)	127 (123–140)	136 (128–149)*	137 (119–148)
Creatinine clearance (mL/min)	49 (21–129)	75 (25–115)	84 (40–135)*
Plasma osmolarity (mosm/kg)	308 (261–329)	305 (278–317)	287 (254–316)
Calc. osmolarity (mosm/kg)	298 (259–313)	298 (259–313)	282 (245–310)
Blood urea nitrogen (mmol/L)	15.2 (4.3–52)	9.8 (3.2–27.9)*	4.8 (2.9–12.1)†
UCR‡	28.3 (15–58)	23.9 (10–41)	15 (8–31)†
Osmolar gap	9 (2.5–17.5)	5.1 (−7 to 29)	5 (0–22)
Urine osmolarity (mosm/kg)	573 (135–826)	585 (240–939)	432 (246–986)
Urinary sodium (mmol/L)	31.5 (10–66)	75.5 (9–143)	108 (32–241)†
Plasma potassium (mmol/L)	4.6 (3.3–5.3)	3.9 (3.4–5.5)	4 (2.4–4.6)*
Fractional excretion of sodium	0.54 (0.05–1.26)	0.8 (0.06–5.48)	–

All values are medians (range).

* *P* < 0.05 and

† *P* < 0.01 versus admission.

‡ Serum urine: creatinine ratio non-SI units.
